# Investigating Global Spatial Patterns of Diarrhea-Related Mortality in Children Under Five

**DOI:** 10.3389/fpubh.2022.861629

**Published:** 2022-07-15

**Authors:** Ali Almasi, Alireza Zangeneh, Arash Ziapour, Shahram Saeidi, Raziyeh Teimouri, Tohid Ahmadi, Mehdi Khezeli, Ghobad Moradi, Moslem Soofi, Yahya Salimi, Nader Rajabi-Gilan, Seyed Ramin Ghasemi, Fatemeh Heydarpour, Shahrzad Moghadam, Tan Yigitcanlar

**Affiliations:** ^1^Social Development and Health Promotion Research Center, Health Institute, Kermanshah University of Medical Sciences, Kermanshah, Iran; ^2^Cardiovascular Research Center, Health Institute, Kermanshah University of Medical Sciences, Kermanshah, Iran; ^3^Department of Art, Architecture, and Design, University of South Australia, Adelaide, SA, Australia; ^4^Geography and Urban Planning, Academic Member of Academic Center for Education, Culture and Research (ACECR), Shahid Beheshti University of Tehran, Tehran, Iran; ^5^Social Determinants of Health Research Center, Research Institute for Health Development, Kurdistan University of Medical Sciences, Sanandaj, Iran; ^6^Research Center for Environmental Determinants of Health (RCEDH), Health Institute, Kermanshah University of Medical Sciences, Kermanshah, Iran; ^7^Medical Biology Research Center, Health Technology Institute, Kermanshah University of Medical Sciences, Kermanshah, Iran; ^8^Geography and Urban Planning, University of Zanjan, Zanjan, Iran; ^9^School of Architecture and Built Environment, Queensland University of Technology, Brisbane, QLD, Australia

**Keywords:** spatial pattern, child death, diarrheal diseases, GIS, global

## Abstract

**Objective:**

Investigating the trends of child diarrhea-related mortality (DRM) is crucial to tracking and monitoring the progress of its prevention and control efforts worldwide. This study explores the spatial patterns of diarrhea-related mortality in children under five for monitoring and designing effective intervention programs.

**Methods:**

The data used in this study was obtained from the World Health Organization (WHO) public dataset that contained data from 195 countries from the year 2000 to 2017. This dataset contained 13,541,989 DRM cases. The worldwide spatial pattern of DRM was analyzed at the country level utilizing geographic information system (GIS) software. Moran's I, Getis-Ord Gi, Mean center, and Standard Deviational Ellipse (SDE) techniques were used to conduct the spatial analysis.

**Results:**

The spatial pattern of DRM was clustered all across the world during the study period from 2000 to 2017. The results revealed that Asian and African countries had the highest incidence of DRM worldwide. The findings from the spatial modeling also revealed that the focal point of death from diarrhea was mainly in Asian countries until 2010, and this focus shifted to Africa in 2011.

**Conclusion:**

DRM is common among children who live in Asia and Africa. These concentrations may also be due to differences in knowledge, attitude, and practices regarding diarrhea. Through GIS analysis, the study was able to map the distribution of DRM in temporal and spatial dimensions and identify the hotspots of DRM across the globe.

## Introduction

Diarrhea is a common symptom of a gastrointestinal infection caused by a variety of bacterial, viral, and parasitic organisms and is evidenced by loose, watery, and increased frequency of stools when infected. Poor hygienic standards cause the infection which is transmitted through food and water contaminated by infectious persons. When those who are malnourished or have poor immunity get chronic diarrhea or severe diarrhea, it can lead to dehydration and potentially even death (1). Diarrhea has been a major challenge and an important concern for public health because it is the leading cause of death in some parts of the world (2, 3). Considering that diarrhea results in malnutrition in children, every year 1.7 billion children suffer from diarrhea all over the world. Despite preventive measures, it still is the second leading cause of death among children (4). Previous studies indicate that, although it is a major challenge that continues to exist, mortality due to diarrhea remains largely avoidable and renewed efforts are urgently required to reduce the disease burden (5).

Diarrhea-related mortality (DRM) is prevalent globally in all regions. However, a disproportionate number of DRMs occur in low-income countries, which have fewer resources and less sturdy urban and health infrastructure to manage the burden than high-income countries (5). Around the world, many people still have no access to potable water and adequate sanitation. In low-income countries, children under 3 years of age experience a mean figure of three times diarrhea every year. Each instance of diarrhea denies the child nutrition essential for growth (4).

Geographic information systems (GIS) have been used as a tool for investigating a range of diseases related to environmental issues (6, 7). GIS can offer an efficient and practical way to directly visualize the dynamics of disease transmission and identify the geographic distribution and risk factors of epidemic outbreaks. It has been used in creating spatial patterns of relationships between local factors and disease occurrences in people (8). Thus, GIS and spatial analysis may help explain DRM in children under five and its causes over a given area. Spatial analysis may be used to explore the co-occurrence of a health event and other population events in the same geographic unit (9).

In recent years, the epidemiology of DRM has been changing. There is an observable decline in mortality, particularly among children younger than 5 years. Increased access to the rotavirus vaccine, improvement in child growth and wellbeing, and provision of universal access to safe water and sanitation has been very helpful in further reducing the burden of preventable diseases like diarrhea (10). In identifying the magnitude of this burden, the global health community has made prevention and treatment of DRM a priority (5). Policymakers have formulated various global action schemes for the health of women and children, particularly to address child mortality, many of which have been successful (1). Nonetheless, epidemiological evidence shows that there is a research gap in largely avoidable DRMs, and further efforts to decrease the disease burden are urgently needed (5). Literature reveals that DRMs have been investigated primarily at local and regional levels (2, 5, 11, 12). There are no prior research studies, to our knowledge, on the global spatial distribution of deaths caused by diarrheal diseases. Our study focuses on filling this gap in the literature. This study, therefore, is a pioneering effort aimed at examining the spatial pattern of DRM in children under five across the globe.

## Methods

### Study Design and Dataset

The data used in this study was obtained from the World Health Organization (WHO) which had public data from 195 countries for the period, 2000–2017 (http://apps.who.int/gho/data/view.main.ghe1002015-CH3?lang=en). The raw data was available at the country level, and the analysis has been conducted at the country level.

We determined the modeling strategy for each country based on the quality and availability of data. This study analyzed the DRM information of 13,541,989 children under five. The worldwide spatial pattern of deaths caused by diarrheal diseases was analyzed using GIS and Moran's I, Getis-Ord Gi, Mean Center, and Standard Deviational Ellipse (SDE).

### The Spatial Autocorrelation (Global Moran's I) and Hot Spot Analysis (Getis-Ord Gi^*^)

The global spatial autocorrelation statistical methodology was used to measure the correlations in regional observations, find patterns, and determine the levels of spatial clustering among neighboring countries (13). Also, we utilized the spatial weight matrix construction in spatial autocorrelation for data analysis as a spatial weights matrix file (.swm). It compared the values of variables in a single location with those in other locations (14, 15). It was calculated as follows:


(1) $$\begin{array}{l} Equation\left(1\right):\;I=\frac{N\sum_{i}\sum_{j}w_{i.j}\left(X_{i}-\overset{-}{X}\right)\left(X_{j}-\overset{-}{X}\right)}{\left(\sum_{i}\sum_{j}w_{i.j}\right)\sum_{i}\left(X_{i}-\overset{-}{X}\right){\left(X_{j}-\overset{-}{X}\right)}^{2}} \end{array}$$

Where *N* was the number of patients, *Xi* was the value of the variable in a certain location, *Xj* was the value of the variable in another location, *X* was the mean of the variable, and *Wij* was the weight used for comparing locations *i* and *j*. *Wij* was a weighted matrix based on distance, and it was the reversed distance between locations *i* and *j* (16).

When values for two neighboring features are either both larger than the mean or both smaller than the mean, the cross-product will be positive. When one value is smaller than the mean and the other value larger, then the cross-product will be negative. In all instances, the higher the deflection from the average, the larger the cross-product effect. If the values in the dataset tend to cluster spatially (high values cluster near other high values; low values cluster near other low values), the Moran's Index will be positive. When high values repel other high values and tend to be near low values, the Index will be negative. If positive cross-product values balance negative cross-product values, the Index will be near zero. The numerator is normalized by the variance so that Index values fall between −1.0 and +1.0. The local Gi^*^(d) statistic (local G-statistic) is used to test the statistical significance of localized clusters (i.e., related to leading causes of death), and to establish the spatial extent of these clusters (17). The local G-statistic is useful for identifying individual members of local clusters by determining the spatial dependence and relative magnitude between an observation and its neighboring observations (18). The equation for calculating the local G-statistic is as follows:


(2) $$\begin{array}{l} Equation(2):G\overset{*}{i}\left(d\right)=\frac{\sum jWij\left(d\right)\underset{j}{X}-\text{W*i }\overset{-}{X^{*}}}{\overset{*}{S}\{[(nS^{*}1i)-W_{i}^{*2}]/\left(n-1\right)\}½} \end{array}$$

**Table 1:** The Global Moran's I for DRM in children under five worldwide in the years 2000–2017.

**Years**	**Moran's I**	**Z-score**	**Pvalue**	**Interpretation**
2000	0.05	3.12	0.00	Clustered
2001	0.05	3.10	0.00	Clustered
2002	0.05	3.07	0.00	Clustered
2003	0.05	3.07	0.00	Clustered
2004	0.05	3.06	0.00	Clustered
2005	0.05	3.05	0.00	Clustered
2006	0.05	3.03	0.00	Clustered
2007	0.05	3.04	0.00	Clustered
2008	0.05	3.10	0.00	Clustered
2009	0.05	3.16	0.00	Clustered
2010	0.06	3.18	0.00	Clustered
2011	0.06	3.24	0.00	Clustered
2012	0.06	3.30	0.00	Clustered
2013	0.06	3.33	0.00	Clustered
2014	0.06	3.32	0.00	Clustered
2015	0.06	3.25	0.00	Clustered
2016	0.06	3.25	0.00	Clustered
2017	0.06	3.27	0.00	Clustered

Where *Wij (d)* is the spatial weight vector that defines values for location *j* (within the distance of *d*) from neighboring location *i*. W^*^i is the sum of the weights *Wij*, S^*^1i is the square sum of the weights, and S^*^ is the standard deviation of data in the locations and, *n* is the number of cases (16).

Getis-Ord Gi^*^ recognizes statistically significant spatial clusters of high values (hotspots) and low values (cold spots). It creates an Output Feature Class with a z-score, *p*-value, and confidence level bin (Gi_Bin) for each feature in the Input Feature Class.

### Mean Center

The Mean Center is the average *x* coordinate and *y* coordinate for all features in the geographical area being studied. It is useful for tracking changes in the diffusion or for comparing the diffusions of various types of features (16):

It is determined by:


(3) $$\begin{array}{l} \overset{-}{X}=\frac{\overset{n}{\underset{i=1}{\sum }}x_{i}}{n},\overset{-}{Y}=\frac{\overset{n}{\underset{i=1}{\sum }}y_{i}}{n} \end{array}$$

Where *x*_*i*_ and *y*_*i*_ are the peculiarities for distinguishing feature *i*, and *n* is the sum of the feature.

The Weighted Mean Center is calculated using:


(4) $$\begin{array}{l} \overset{-}{X_{w}}=\frac{\overset{n}{\underset{i=1}{\sum }}w_{i}x_{i}}{\overset{n}{\underset{i=1}{\sum }}w_{i}},\overset{-}{Y_{w}}=\frac{\overset{n}{\underset{i=1}{\sum }}w_{i}y_{i}}{\overset{n}{\underset{i=1}{\sum }}w_{i}} \end{array}$$

Where *w*_*i*_is the weight of distinguishing feature*i*.

Furthermore, to calculate the center (c) for a 3rd aspect if a *z* attribute exists for each distinguishing feature, the following equation is used:


(5) $$\begin{array}{l} \overset{-}{Z}=\frac{\overset{n}{\underset{i=1}{\sum }}z_{i}}{n},\overset{\;}{Z_{w}=\frac{\overset{n}{\underset{i=1}{\sum }}w_{i}z_{i}}{\overset{n}{\underset{i=1}{\sum }}w_{i}}} \end{array}$$

### Standard Deviational Ellipse

A usual way of measuring the trend of a prevalent disease among the population is to calculate the standard distance separately in the *x* and *y* coordinates. These two measures define the axis of an ellipse encompassing the distribution of features. It is referred to as the Standard Deviational Ellipse (SDE), as the method calculates the standard deviation of the *x* coordinates and *y* coordinates from the Mean Center to define the axis of the ellipse. The ellipse allows us to see if the distribution of the features is elongated and determine if it has a particular orientation. Graphing ellipses for disease-related deaths such as diarrhea surveillance programs can, over time, potentially offer real-time outcomes of its spatial spread trend, as researchers continue their focus on the central trends and diffusion patterns.

The SDE is determined by:


(6) $$\begin{array}{l} Equation\left(3\right):SDE_{x}=\frac{\overset{n}{\underset{i=1}{\sum }}{\left(x_{i}-\overset{-}{X}\right)}^{2}}{n},SDE_{y}=\sqrt{\frac{\overset{n}{\underset{i=1}{\sum }}{\left(y_{i}-\overset{-}{Y}\right)}^{2}}{n}} \end{array}$$

Where *x*_*i*_ and *y*_*i*_ are the coordinates for feature *i*, $\left\{\overset{-}{X},\overset{-}{Y}\right\}$ represents the Mean Center for the features, and n is the sum of the feature.

The angle of rotation is calculated as:


(7) $$\begin{array}{l} \tan\theta =\frac{A+B}{C} \end{array}$$

(8) $$\begin{array}{l} A=\left(\overset{\;}{\underset{i=1}{\sum }}\overset{\;}{\overset{-}{x_{i}^{2}}-\overset{n}{\underset{i=1}{\sum }}\overset{-}{y_{i}^{2}}}\right) \end{array}$$

(9) $$\begin{array}{l} B=\sqrt{{\left(\overset{n}{\underset{i=1}{\sum }}\overset{-}{x_{i}^{2}}-\overset{n}{\underset{i=1}{\sum }}\overset{-}{y_{i}^{2}}\right)}^{2}+4{\left(\overset{n}{\underset{i=1}{\sum }}\overset{\;}{{\overset{-}{x}}_{i}\overset{-}{y_{i}}}\right)}^{2}} \end{array}$$

(10) $$\begin{array}{l} C=2\overset{n}{\underset{i=1}{\sum }}\overset{-}{x_{i}}\overset{-}{y_{i}} \end{array}$$

Where ${\overset{-}{x}}_{i}$and $\overset{-}{y_{i}}$are the deviations of the *xy*coordinates from the Mean Center.

The standard deviations for the *x* axis and *y*axis are:


(11) $$\begin{array}{l} \sigma_{x}=\sqrt{2}\sqrt{\frac{\overset{n}{\underset{i=1}{\sum }}{\left(\overset{-}{x_{i}}\cos\theta -\overset{\;}{{\overset{-}{y}}_{i}\sin\theta }\right)}^{2}}{n}} \end{array}$$

(12) $$\begin{array}{l} \sigma_{y}=\sqrt{2}\sqrt{\frac{\overset{n}{\underset{i=1}{\sum }}{\left(\overset{-}{x_{i}}\sin\theta -\overset{\;}{{\overset{-}{y}}_{i}\cos\theta }\right)}^{2}}{n}} \end{array}$$

## Results

### Global Moran's I for DRM in Years 2000–2017

**Figure 1:**
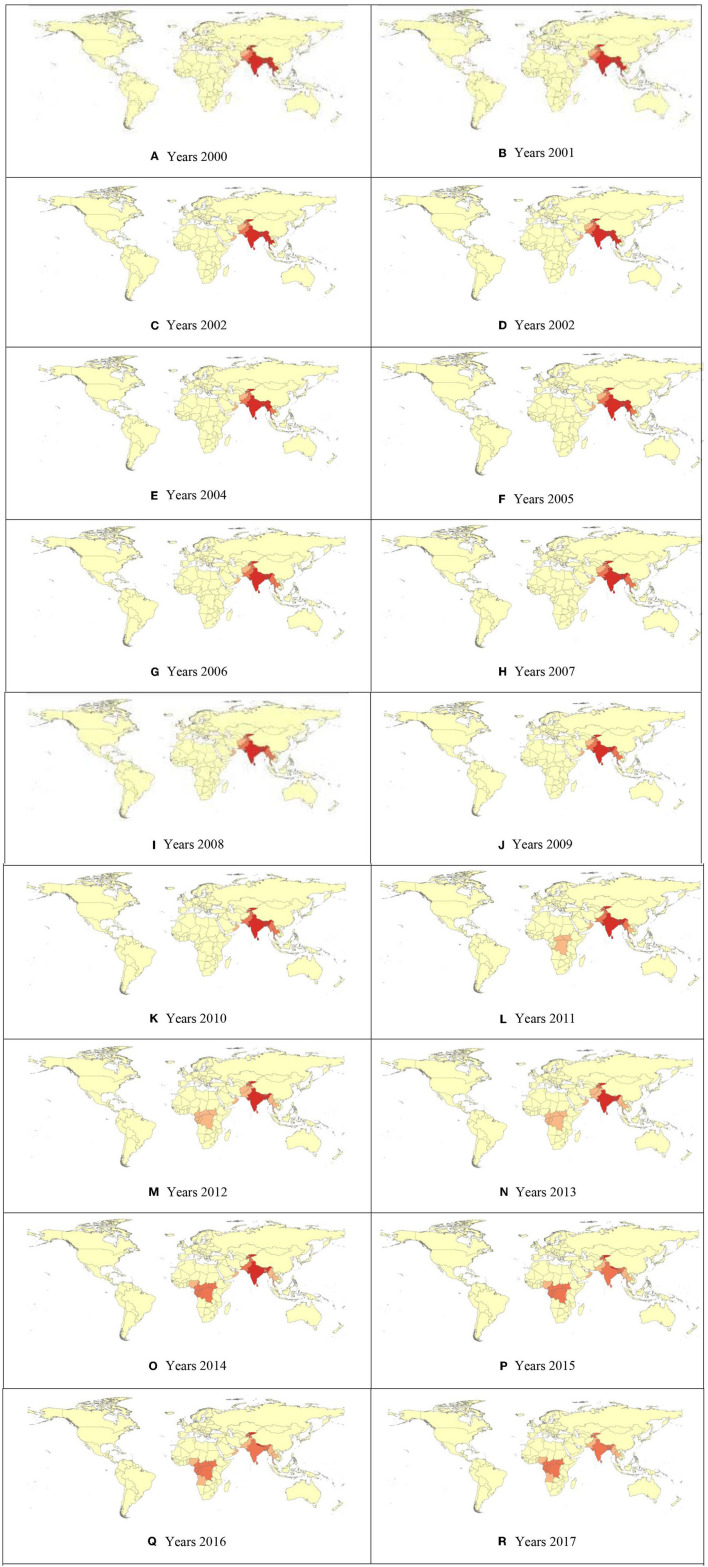
**(A–R)** Hot Spot Analysis (Getis-Ord Gi*) of Child Mortality due to diarrheal diseases Gi_Bin.

The Moran's I value of 0.05 (*p* < 0.001) for the year 2000 demonstrated that there was a notable clustering of DRM in children under five all over the world. A z-score of 3.12 confirmed that there was less than a 1% probability that this clustered pattern of DRM could be entirely the result of random chance Figure 1A. Therefore, it was widely prevalent in 195 countries worldwide. The results also showed that Moran's I for the clustering process during the period 2000–2017 increased from 0.05 to 0.06 (Figure 1). This indicated the formation of highly concentrated clusters of deaths from diarrheal disease worldwide (Figure 1).

### Getis-Ord Gi^*^, Mean Center, and SDE

**Figure 2:**
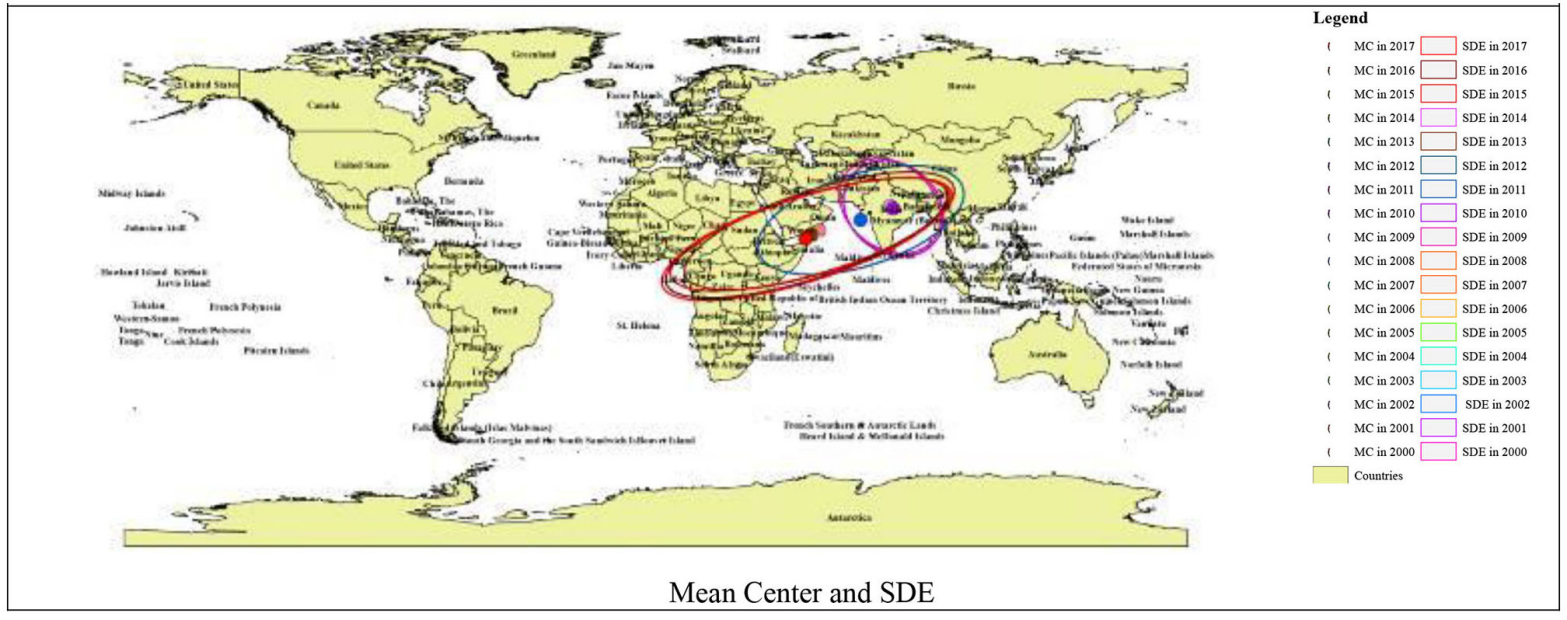
Spatial clustering of diffused DRM, Getis Ord Gi* hotspot cluster analysis, Mean Center, and SDE.

The Getis-Ord Gi^*^ hotspot analysis recognized neighboring hotspots in Asia and Africa. The DRM in children under five was not randomly distributed (Figure 2). The overlap of the cluster areas of diffused DRM was noted, pointing perhaps to a co-factor in the area.

Figure 2 shows the DRM's Mean Center calculated for the world countries from 2000 to 2017. The results demonstrated that the Mean Center was in India for the years 2000 to 2010, and included most Asian countries (India, Nepal, Bangladesh, Myanmar, Afghanistan, Yemen, Uzbekistan, Kazakhstan, and Sri Lanka). The Standard Deviational Ellipse also had a northwest-southeast direction. However, from 2011 onwards, the average DRM has shifted to African countries (Nigeria, Cameroon, Equatorial Guinea, Gabon, Republic of the Congo, the Democratic Republic of the Congo, and Uganda), and the SDE has similarly shifted southwest-northeast (Figure 2).

## Discussion

In this study, we examined the spatial pattern of DRMs of children under five across the globe from 2000 to 2017 using GIS analysis.

### Clustering of Spatial Patterns

This analysis showed clustered patterns in the distribution of DRMs across the world during the study period (2000–2017) (Figure 1). The results of other studies in Chennai (Chennai Corporation, Tamilnadu, India) (19) and rural areas of Bangladesh (12) also showed the clustered spatial pattern of DRMs. The results of the analysis showed that environmental factors such as contaminated water and food caused diarrhea and consequently led to mortality (20). Additionally, human factors such as social structures, lifestyles, race, culture, religion, immigration, and politics have also been factors contributing to DRMs, (21–25) which may have been the reason for the clustering of disease-related mortality worldwide. Cluster formation of disease-related mortality confirms that health issues always have spatial dimensions (26–30). Hence, it is recommended that health policymakers and planners study the causes of the formation of these hotspots.

### Higher Risk of Mortality

The current study documents that Asian and African countries had the highest levels of DRM during the study period (2000–2017) (Figure 2). Diarrhea has been responsible for the deaths of more than 90% of children under five in low and lower-middle income countries. Regionally, South Asia and sub-Saharan Africa accounted for 88% of deaths in the same age group. The results of studies in India (11, 19) and Africa done at the local level in these countries are also similar to our study findings (31). These high levels of DRMs, as stated in other studies, are likely because of the socio-economic and cultural environments ensuing in the countries that have fewer resources and limited infrastructure to bear the disease burden (32, 33). Children living in deprived or isolated communities are particularly vulnerable to health hazards. In this regard, the result of the analysis showed that children died from preventable diseases because effective interventions were not provided equitably to all communities (1). Thus, children who are undernourished or have impaired immunity and people living with HIV have been most at risk of life-threatening diarrhea (4). Furthermore, variations in the impact of diarrhea among different communities might be due to diversity in awareness, attitude, and practices regarding diarrheal diseases. Besides, a feasible reason might also be due to environmental and infrastructural disparities such as access to water, availability and utilization of toilet facilities, access to hand washing facilities, and ways of waste disposal (34). Therefore, there is a need to increase the availability and accessibility of these facilities in targeted areas through a unified and comprehensive approach to reducing diarrhea-related morbidity and mortality among children under five (35). In some of the Asian and African counties that have the highest levels of DRM, due to poverty, undernutrition, poor hygiene, congested households with inadequate or no sanitation, and absence of proper care (10), children in these conditions are highly vulnerable to infections including diarrhea. In contrast, diarrhea-related mortality among children in developed countries has decreased in recent decades due to successful improvements in sanitation, hygiene, and access to antibiotics (9).

This study also found that DRM in children under five was prevalent in Asian countries until 2010 and has since shifted to African countries (Figure 2). The findings of our study confirm the theory of spatial distribution. According to the theory of spatial distribution, this relocation is probably influenced by environmental conditions and complex cultural and socio-economic processes (34, 35). However, this situation requires further investigation as emphasized by the WHO (4). Therefore, further research is recommended in this regard.

### Tools for Policymakers

Our study indicated that GIS can be an effective tool for policymakers to identify changes in DRM worldwide. The results of other studies have also emphasized the efficacy of GIS to assess the risk of diarrhea in other regions (36, 37). GIS can be effective in understanding the distribution pathways, the trend of the disease spread, and their relationship to environmental factors (such as climatic conditions, water quality, health status, agricultural and industrial activities, and environmental contaminants), and assist in investigating the causes of diarrhea and its mortality. Moreover, the results from other studies reveal that a significant proportion of diarrheal diseases can be prevented through the provision of clean drinking water and adequate sanitation and hygiene (4). Therefore, we recommend GIS analysis for the effective and timely identification of DRM hotspots.

### Strength

This research has mapped the incidence of DRM across the globe and generated fresh insights into the identification of DRM hotspots. The approach introduced in this paper can generate informed decision-making opportunities for policymakers and program planners. Besides, further spatial analysis of the identified hotspot areas by incorporating other factors such as health, environment, socioeconomic, and infrastructure could help health professionals to be prepared for the challenges ahead and develop customized intervention programs in the identified hotspots.

### Limitations

This study had several limitations. First, DRM could be affected by climatic factors such as rainfall, temperature, humidity, and so on, which we were unable to explore. Second, we could not examine the spatial pattern of DRM by gender. Third, we also did not have access to social variables and other lifestyle factors including socioeconomic status, drinking water supply, and toilet facilities that could have an impact on DRMs. Fourth, we only explored DRM globally and were unable to explore mortality from diarrhea in underdeveloped, developing, or developed/advanced countries separately.

## Conclusion

This study indicates clustered patterns in the distribution of DRM in the world during the period 2000–2017, where Asian and African countries had the highest levels of DRM. Nevertheless, notably, from 2011 onwards, new and more dangerous hotspots have emerged in Africa. Through GIS analysis, we were able to map the spatial distribution of DRM in temporal and spatial conditions and identify the hotspots of DRM worldwide.

## Data Availability Statement

Publicly available datasets were analyzed in this study. This data can be found here: http://apps.who.int/gho/data/view.main.ghe1002015-CH3?lang=en.

## Ethics Statement

All necessary permissions were obtained. The data used in this study came from the WHO public data of 195 countries during 2000–2017 to access and use the data. This study was approved by the Scientific Ethics Committee of the Kermanshah University of Medical Sciences. Reference Number: IR.KUMS.REC.1398.575.

## Author Contributions

AA, AZa, SS, and RT contributed to the study design, data collection, analysis, and write-up. TA contributed to data analysis and write-up. GM, MS, YS, NR-G, MK, SR, AZi, and TY contributed to the study design and data analysis. AA, AZa, SM, SS, RT, and TY contributed to the study design and write-up. All authors read and approved the final version of the manuscript. All authors contributed to the article and approved the submitted version.

## Funding

This work was supported by colleagues in the Kermanshah University of Medical Sciences, as well as the financial and spiritual support of the deputy head of the Research and Technology Department of the Kermanshah University of Medical Sciences under Grant (Number 980462). This project benefited from data processing and analysis and research design by the Kermanshah University of Medical Sciences, Iran.

## Conflict of Interest

The authors declare that the research was conducted in the absence of any commercial or financial relationships that could be construed as a potential conflict of interest.

## Publisher's Note

All claims expressed in this article are solely those of the authors and do not necessarily represent those of their affiliated organizations, or those of the publisher, the editors and the reviewers. Any product that may be evaluated in this article, or claim that may be made by its manufacturer, is not guaranteed or endorsed by the publisher.
